# Silencing IGFBP-2 decreases pancreatic cancer metastasis and enhances chemotherapeutic sensitivity

**DOI:** 10.18632/oncotarget.18669

**Published:** 2017-06-27

**Authors:** Huan Liu, Le Li, Hua Chen, Rui Kong, Shangha Pan, Jisheng Hu, Yongwei Wang, Yilong Li, Bei Sun

**Affiliations:** ^1^ Department of Pancreatic and Biliary Surgery, The First Affiliated Hospital of Harbin Medical University, Harbin, Heilongjiang, China

**Keywords:** IGFBP-2, pancreatic cancer, metastasis, Hedgehog, gemcitabine

## Abstract

Pancreatic cancer has remained one of the most devastating and lethal malignancies characterized by local invasion, distant metastasis and a high degree of chemoresistance. Insulin-like growth factor binding protein 2 (IGFBP-2) is a member of the IGFBP family of proteins, and it is highly expressed in pancreatic cancer patients’ serum and tumor tissues. IGFBP-2 also mediates tumor cell growth, invasion and resistance, while the mechanisms remain unclear. In this study, we sought to determine the impact of IGFBP-2 expression on pancreatic cancer tumorigenesis and metastasis *in vitro* and *in vivo*. Wound healing, migration and invasion assays revealed that knockdown of IGFBP-2 inhibits cancer cell migration and invasion. Downregulation of IGFBP-2 attenuates EMT via increasing the E-cadherin and reducing the vimentin and N-cadherin. PTCH-1 is found contribute to the function of IGFBP-2 in suppressing metastasis and EMT of pancreatic cancer. Silencing IGFBP-2 inhibited invasion and metastatic properties, partially through inhibiting PTCH1 in pancreatic cancer. Additionally, inhibition of IGFBP-2 enhanced the sensitivity of pancreatic cancer cells to gemcitabine, suppressed tumor growth and potentiated the anti-tumor effect of gemcitabine in the orthotopic tumor model. Our results provide novel insight of IGFBP-2 as a promising target to inhibit the metastasis and overcome the chemoresistance in pancreatic cancer.

## INTRODUCTION

Pancreatic cancer is one of the most lethal tumors, with a five-year survival rate of 8% [1]. In contrast to the steady increase in survival for most cancers, advances have been slowed for pancreatic cancers. Pancreatic ductal adenocarcinoma (PDAC) has a poor prognosis largely due to its propensity for early local invasion, distant metastasis and lack of effective therapies [2]. It is of paramount importance to understand the mechanisms that contribute to the progression of this disease.

The insulin-like growth factor (IGF) family has been implicated in the progression of PDAC [3]. Insulin-like growth factor binding protein 2 (IGFBP-2), one of six proteins in the IGFBP family, has been proposed as a potential biomarker in pancreatic cancer [4–5]. IGFBP-2 protein is elevated in pancreatic juice and tissue as well as in the plasma of PDAC patients [6–7]. The Hedgehog (Hh) signaling pathway is a ‘core’ signal transduction pathway in pancreatic cancer. The abnormal activation of Hh signaling pathway can lead to the promotion of tumor cell proliferation, migration and invasion [8–10]. Many studies have shown that the main mechanisms of Hh signaling pathway are to promote the epithelial-mesenchymal transition (EMT) process [11–12]. Additionally, growing evidence indicates that elevated IGFBP-2 expression is present in solid tumors and correlates with metastasis. However, few studies have explored the relationship between IGFBP-2 and pancreatic cancer [13]. In addition, pancreatic cancer is insensitive to many chemotherapeutic drugs, including the first-line drug gemcitabine (GEM). IGFBP-2 plays critical roles in resistance to chemotherapy in many malignant tumors, such as breast cancer [14], esophageal adenocarcinoma [15], colon cancer [16], lung cancer [17], prostate cancer [18], glioma [19] and leukemia [20]. Thus, IGFBP-2 may enhance the chemotherapy drug resistance of PDAC cells.

IGFBP-2 is the dark horse in metabolism and cancer, and it is once again in the spotlight [21]. A better understanding of IGFBP-2 will ultimately improve our understanding of this “dark horse” in pancreatic cancer. The present study investigated the effects of IGFBP-2 on pancreatic cancer cell proliferation, migration and invasion and the chemosensitivity.

## RESULTS

### Knockdown of IGFBP-2 inhibits pancreatic cancer cells migration and invasion

**Figure 1:**
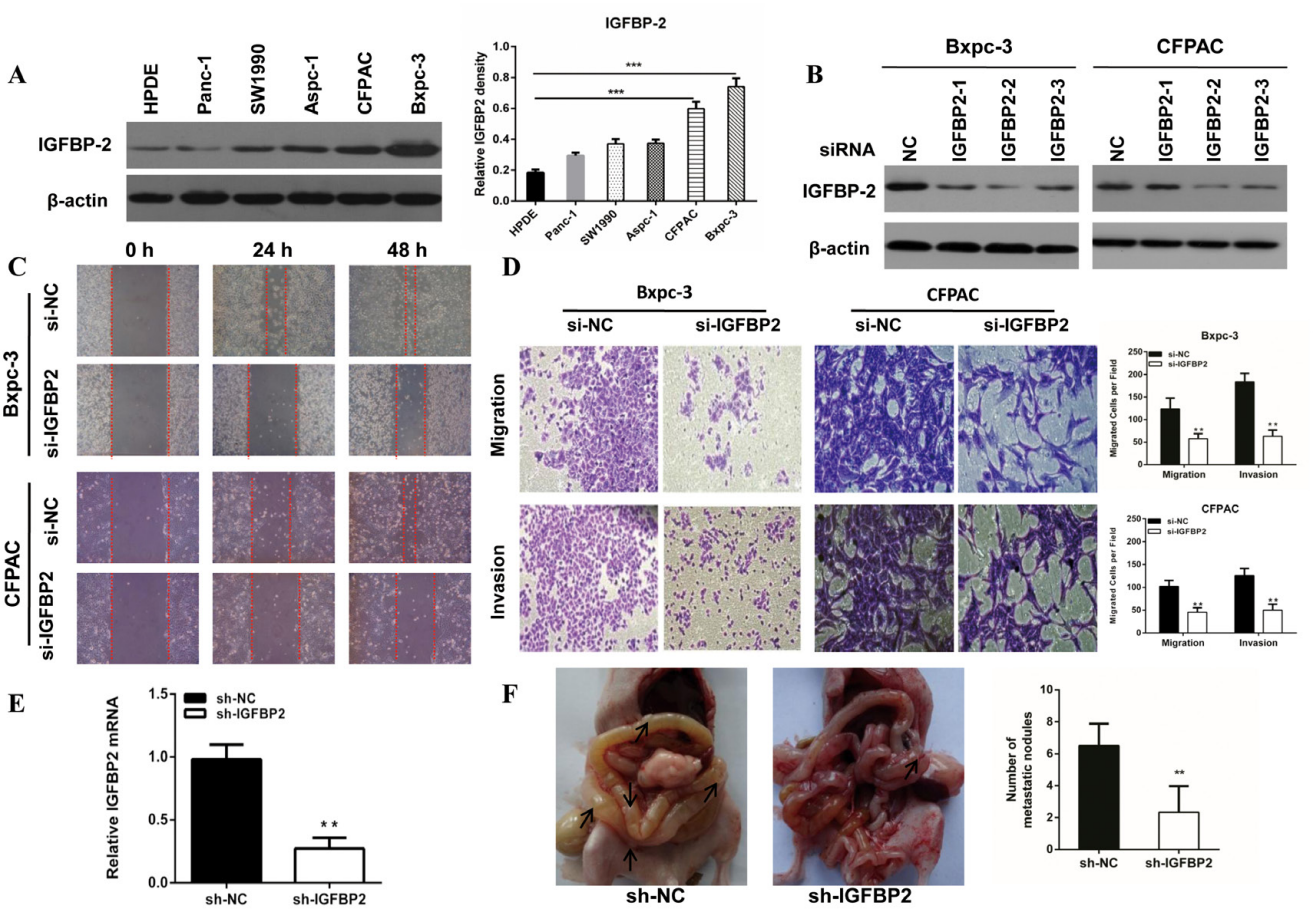
The expression of IGFBP-2 in pancreatic cancer cell lines and knockdown of IGFBP-2 inhibits pancreatic cancer cells migration and invasion **(A)** The expression of IGFBP-2 in Aspc-1, Bxpc-3, CFPAC, Panc-1, SW1990 and HPDE cells as shown by Western blot analysis. Higher level of IGFBP-2 expression was found in Bxpc-3 and CFPAC cells compared with HPDE. **(B)** The expression of IGFBP-2 after treatment with IGFBP-2 siRNA. **(C)** Migratory abilities of Bxpc-3 and CFPAC cells after IGFBP-2 knockdown were recorded for 48h using microscope. **(D)** Representative images of migration and invasion assays for Bxpc-3 and CFPAC cells after IGFBP-2 knockdown were visualized after 48h and 72h, respectively. The number of cells was counted (bottom panel) (Original mignification, 20×). **(E)** IGFBP2 mRNA levels were determined using qRT-PCR. The knockdown rate of sh-IGFBP2 was nearly 70%. **(F)** At day 35, all mice were killed and the metastatic nodules were evaluated. Data was presented as the means±SD of three independent experiments. ** compared with control, *P*<0.01. *** compared with control, *P*<0.001.

To determine the role of IGFBP-2 in pancreatic cancer cell migration and invasion, we compared its expression in five pancreatic cancer lines (Aspc-1, Bxpc-3, CFPAC, Panc-1 and SW1990) with that in HPDE. There was higher expression of IGFBP-2 in Bxpc-3 and CFPAC cells (Figure 1A and 1B).

IGFBP-2 has been demonstrated to be associated with metastasis in various cancers. We investigated its effect on the motility of pancreatic cancer cells using the wound healing and Transwell assays. Our results indicated that IGFBP-2 knockdown cells acquired slower closure of the scratched “wound” compared to the negative control cells (Figure 1C). The transwell assays showed that knockdown of IGFBP-2 markedly decreased migration and invasion capacities in Bxpc-3 and CFPAC cells (Figure 1D). Furthermore, we constructed an orthotopic tumor model to evaluate the role of IGFBP-2 on tumor metastasis *in vivo*. There were less metastasis nodes in the sh-IGFBP2 group compared with the negative control group (Figure 1F). All above results demonstrated that knockdown of IGFBP-2 inhibits migration and invasion of pancreatic cancer cells *in vivo* and *in vitro*.

### Knockdown of IGFBP-2 attenuates EMT of pancreatic cancer cells

**Figure 2:**
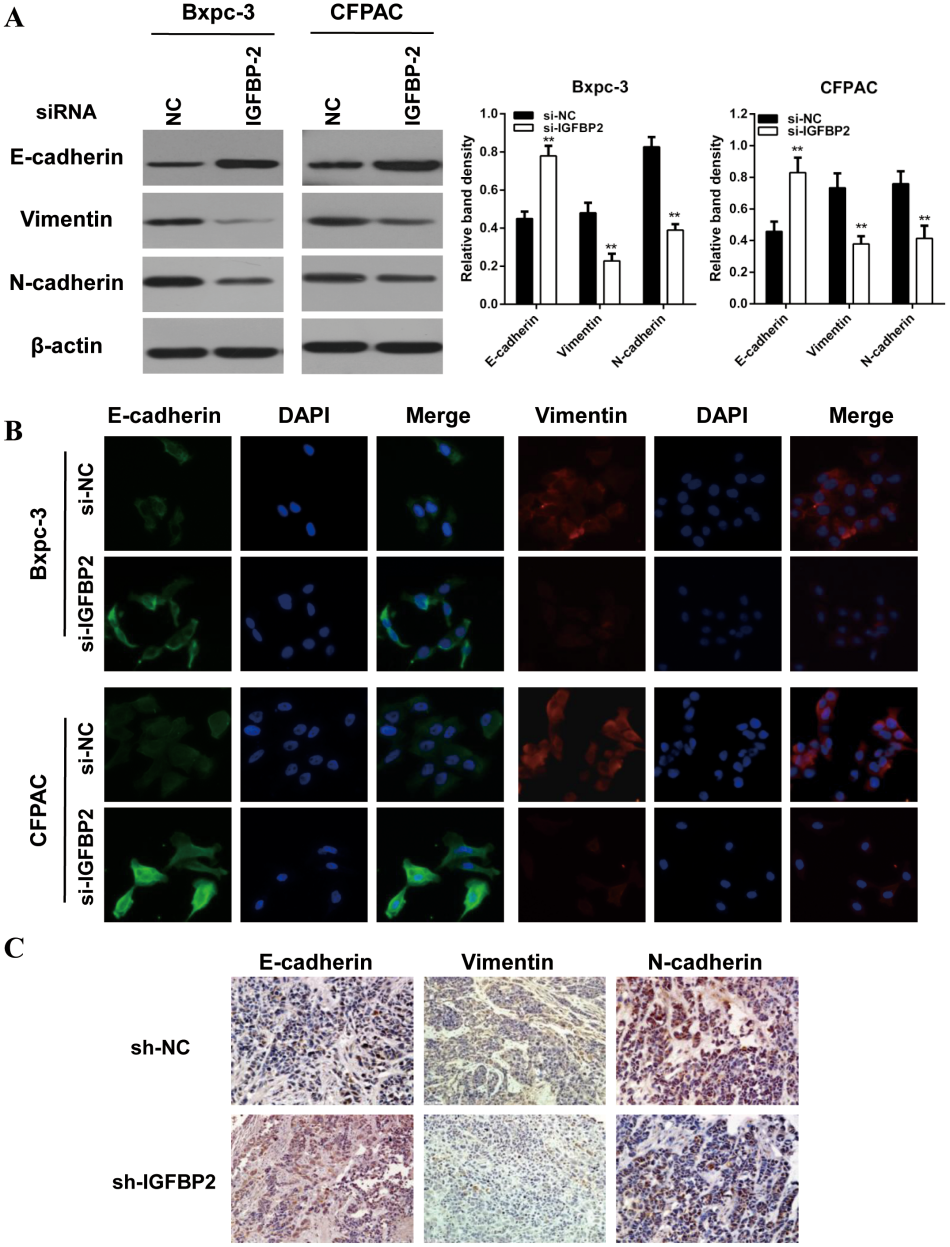
Knockdown of IGFBP-2 attenuates EMT of pancreatic cancer cells **(A)** Western blot showed that knockdown of IGFBP-2 resulted in enhanced expression of the E-cadherin epithelial marker and reduced expression of mesenchymal markers (N-cadherin and vimentin). **(B)** Single and merged images were taken to show immunofluorescence staining of E-cadherin (green) and vimtin (red) accompanied by the cell nucleus (blue) stained by DAPI. Silencing IGFBP-2 elevated the expression of E-cadherin and decreased the expression of vimentin (Original mignification, 20×). **(C)** The expression levels of E-cadherin and N-cadherin were detected by immunohistochemistry in paraffin-embedded tissue sections from the orthotopic pancreatic cancer models (Original mignification, 20×). The results represent means±SD of experiments performed in triplicate. ** compared with control, *P*<0.01.

It has been widely accepted that EMT play pivotal role in pancreatic cancer migration and invasion. Therefore, we investigated the effect of IGFBP-2 on EMT by examining the expression patterns of epithelial and mesenchymal markers. Our results indicated that the epithelial marker (E-cadherin) was increased, whereas the mesenchymal markers (vimentin, N-cadherin) were decreased in IGFBP-2 silenced Bxpc-3 and CFPAC cells (Figure 2A). Similar results were observed in immunofluorescence (IF) staining analysis in Bxpc-3 and CFPAC cells (Figure 2B). Consistent with these morphological data, the immunohistochemistry staining results showed that silencing of IGFBP-2 elevated the expression of E-cadherin and decreased the expression of vimentin and N-cadherin (Figure 2C).

### Knockdown of IGFBP-2 attenuates EMT through the Hedgehog pathway

**Figure 3:**
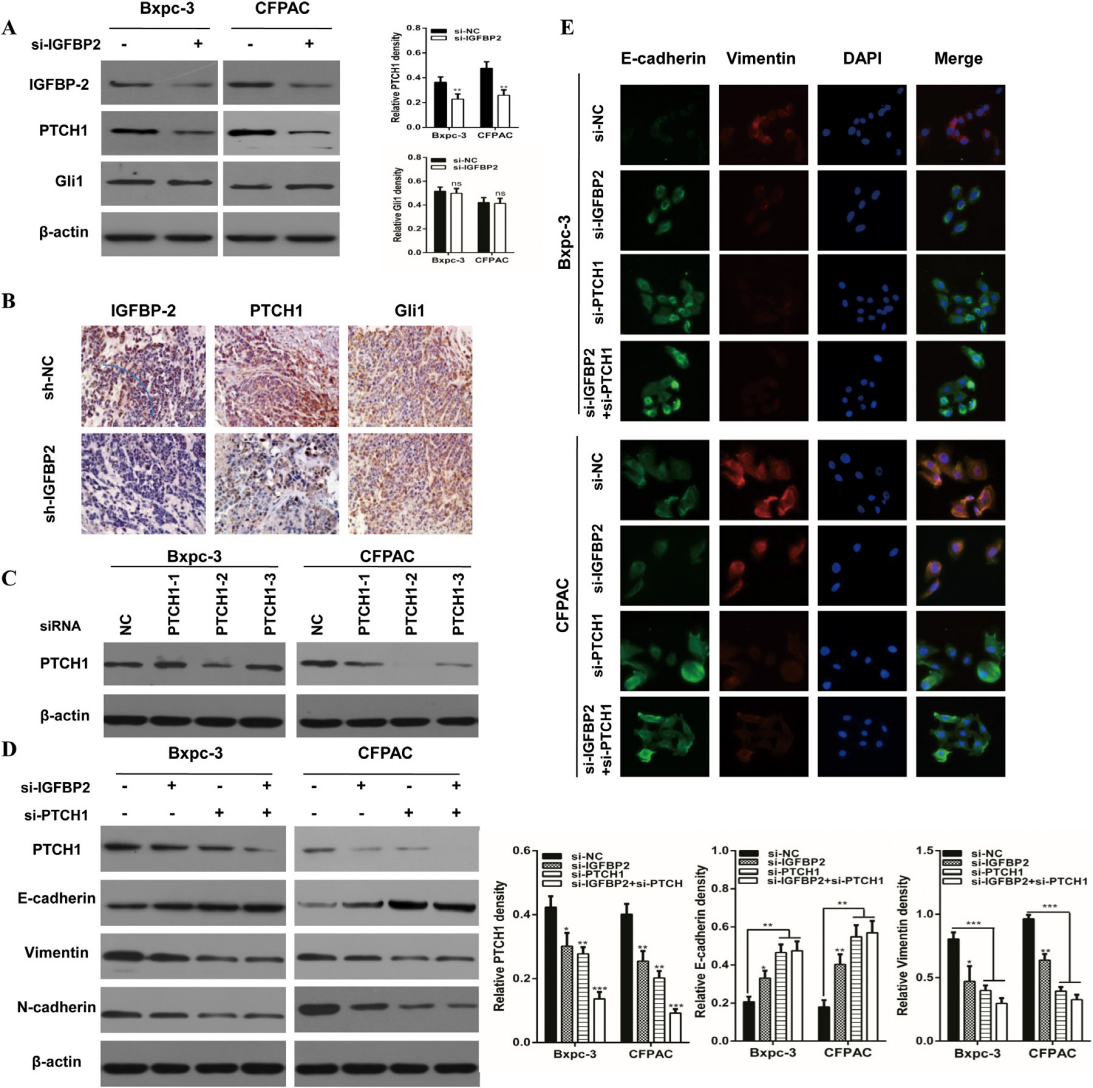
Knockdown of IGFBP-2 attenuates EMT through the Hedgehog pathway by suppressing PTCH1 **(A)** Relative expressions of IGFBP-2, PTCH1 and Gli1 were evaluated by Western blotting in IGFBP-2 knockdown and negative control Bxpc-3 and CFPAC cells. **(B)** The expression of IGFBP-2, PTCH1 and Gli1 was detected by immunohistochemistry in orthotopic tumor tissues (Original mignification, 20×). **(C)** The expression of PTCH1 after treatment with PTCH1 siRNA. **(D)** Western blot assays were underwent in groups of the si-IGFBP2, si-PTCH1 and co-transfected with both si-IGFBP2 and si-PTCH1. The expressions of PTCH1, E-cadherin, vimentin and N-cadherin were detected. **(E)** Immunofluorescence staining showed the expression of E-cadherin and vimentin si-IGFBP2, si-PTCH1 and co-transfected with both si-IGFBP2 and si-PTCH1 (Original mignification, 20×). Data was presented as the means±SD of three independent experiments. ** compared with control, *P*<0.01. *** compared with control, *P*<0.001. ns, not significant.

Genetic analysis of human pancreatic cancers has revealed that a mutation in at least one of the Hedgehog family members is present in 100% of pancreatic cancers, which is one of 12 cellular signaling pathways and processes [22]. Accumulating evidence suggests a number of signaling pathways may be involved in the EMT process, including the TGF-β/Smad, Wnt/β-catenin and Hedgehog pathways [23–25]. IGFBP-2 is a novel target in the Hedgehog pathway [25]. PTCH1 and Gli1 are the key members of the Hh pathway. To investigate whether knockdown of IGFBP-2 attenuates pancreatic cancer EMT through the Hedgehog pathway, they were examined in IGFBP-2 silenced cells. The result indicated that the protein of PTCH1 level was decreased in Bxpc-3 and CFPAC cells silencing IGFBP-2, while Gli1 had no significant change (Figure 3A). Moreover, the immunohistochemical staining results suggested that PTCH1 expression was reduced as a result of IGFBP-2 depletion (Figure 3B).

**Figure 4:**
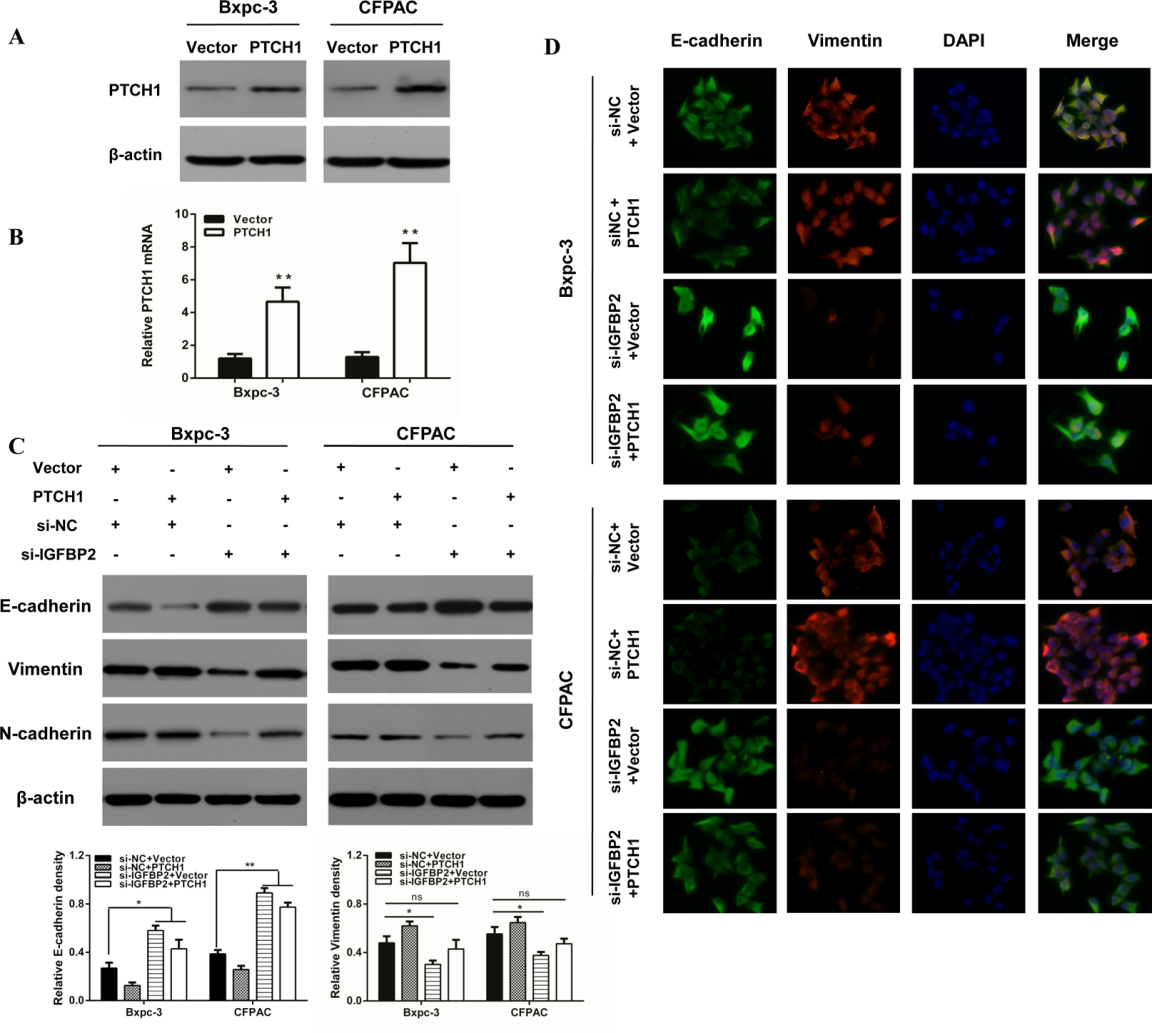
Overexpression of PTCH1 prevents the impact of silencing IGFBP-2 on EMT markers **(A)** Western blot and **(B)** qRT-PCR was performed to verify PTCH1 overexpression. **(C)** Western blot and **(D)** immunofluorescence staining analysis results showed that overexpression of PTCH1 prevents the impact of silencing IGFBP-2 on the expression of epithelial markers (E-cadherin) and the expression of mesenchymal markers (Vimentin and N-cadherin) (Original mignification, 20×). Data was reported as means ± SD for three independent experiments. ** compared with control, *P*<0.01.

Furthermore, activated Gli1 transcription factors translocate to the nucleus and promote transcription of several genes including cyclin D and cyclin E, VEGF, Myc and IGFBP-2 [26]. To further evaluate if PTCH1 is critical for IGFBP-2 mediated promotion of EMT, we utilized the IGFBP-2 silenced cells (Figure 3C). As shown in Figure 3D, PTCH1 protein levels were drastically deceased when si-IGFBP2 and si-PTCH1 were co-transfected. However, when IGFBP-2 and PTCH1 were both silenced, there was no obvious difference in the level of E-cadherin, vimentin and N-cadherin. These results suggest that silencing of PTCH1 does not enhance the effect of IGFBP-2 in EMT process. The same results were found using IF (Figure 3E). To investigate whether overexpression of PTCH1 could prevent the impact of IGFBP-2 on EMT, tumor cells were transfected with si-IGFBP2 and PTCH1 plasmid. Interestingly, Western blot analysis results showed that overexpression of PTCH1 attenuates the effect of IGFBP-2 in EMT process (Figure 4C). Similar results were observed in immunofluorescence staining analysis (Figure 4D). Taken together, these data indicated that knockdown of IGFBP-2 inhibits EMT in pancreatic cancer cells, at least in part, through the Hedgehog pathway by suppressing PTCH1.

### Knockdown of IGFBP-2 inhibits proliferation and increases sensitivity to gemcitabine

**Figure 5:**
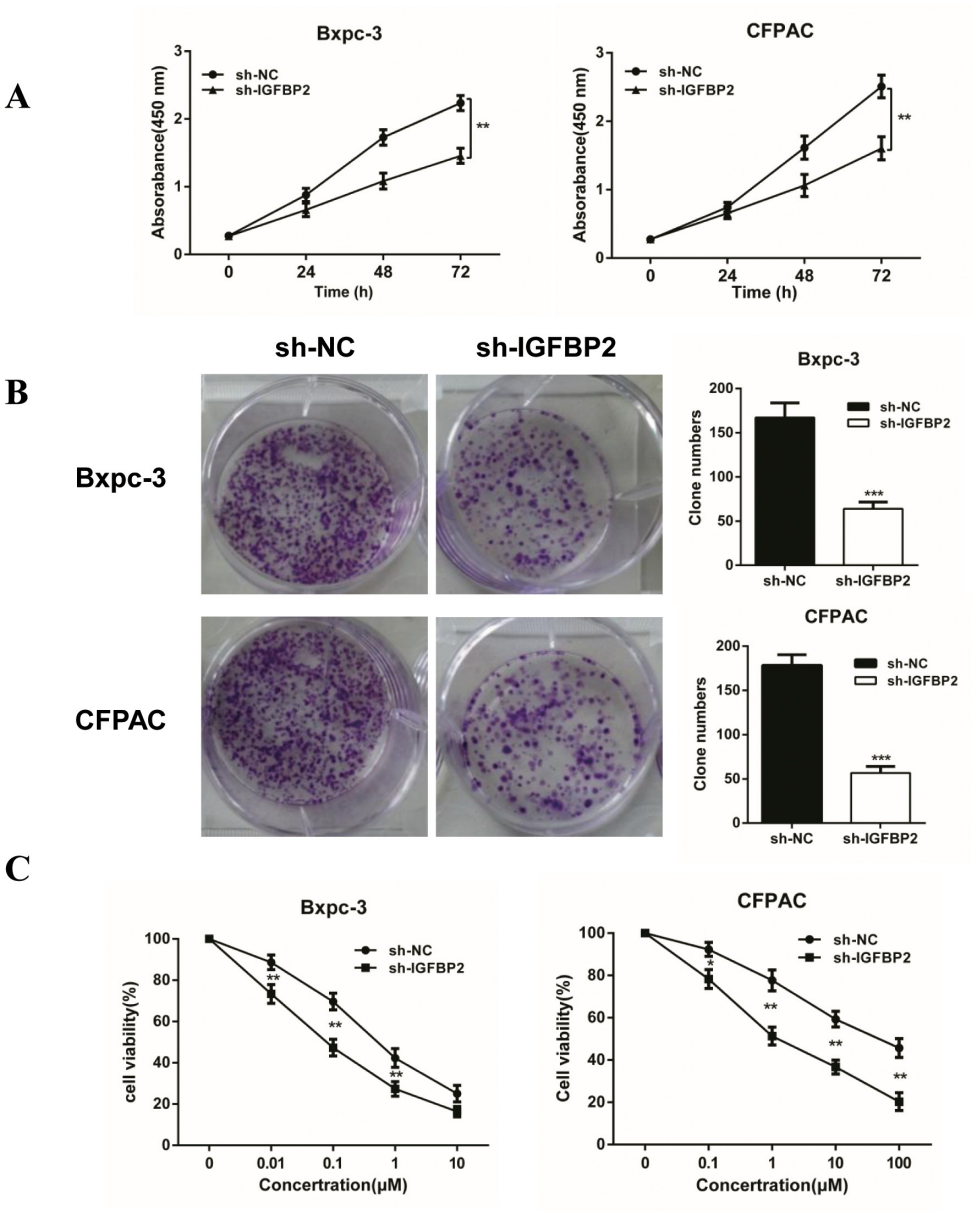
Knockdown of IGFBP-2 inhibits proliferation and increases sensitivity to gemcitabine *in vitro* **(A)** The CCK-8 assay was performed to detect cell proliferation of 24, 48 and 72 h after cells infection. **(B)** Colony formation assays using the Bxpc-3 and CFPAC cell lines are shown for the NC and Lv-IGFBP2 groups. **(C)** The Bxpc-3 and CFPAC cells (NC and Lv-IGFBP2) were incubated with increasing doses of gemcitabine (0-10 μM and 0-100 μM, respectively). The sensitivity of pancreatic cancer cells to gemcitabine was significantly enhanced after IGFBP-2 knockdown. NC, infected with negative lentivirus. Data was reported as means ± SD for three independent experiments. ** compared with control, *P*<0.01. *** compared with control, *P*<0.001.

**Figure 6:**
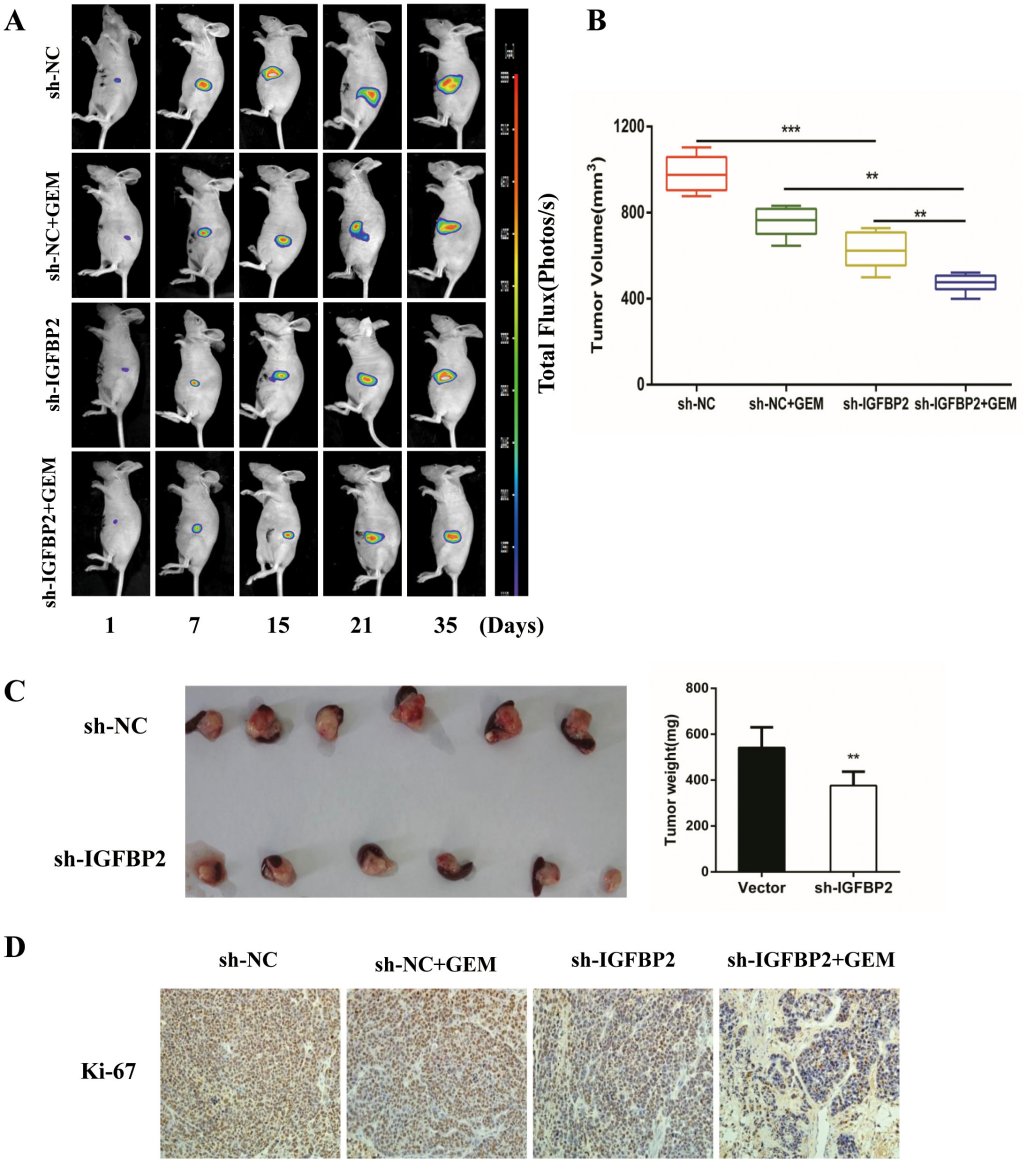
Knockdown of IGFBP-2 suppresses tumor growth and enhances the anti-tumor properties of gemcitabine **(A)** The nude mice of the NC group, the sh-IGFBP2 group, the NC + GEM group and the sh-IGFBP2 + GEM group were bioluminescent imaged at the day of 7, 14, 21, 28 and 35. Representative bioluminescence images of each group are shown. **(B)** The average volumes of orthotopic tumors in the NC group, the sh-IGFBP2 group, the NC + GEM group and the sh-IGFBP2 + GEM group were recorded after 35 days. **(C)** The mice were sacrificed and pancreases were excised at day 35. Representative images of orthotopic tumors are shown above and the weight of tumors is shown on the right. **(D)** Immunohistochemical staining was performed to detect the expression of Ki67 for cell proliferation in tumor tissues. NC, infected with negative lentivirus (Original mignification, 20×). Date was presented as the means ± SD for three independent experiments. *** *P*<0.001.

In addition to the inhibition of pancreatic cancer cell metastasis, IGFBP-2 also has an effect on cell proliferation. CCK-8 and colony formation assays were performed. Knockdown of IGFBP-2 expression led to a decrease in Bxpc-3 and CFPAC cell proliferation at 48 h, with a more significant decrease at 72 h (Figure 5A). Colony formation assays demonstrated that the IGFBP-2 groups yielded less and smaller colonies compared with the negative control groups (Figure 5B). The volume and the weight of the tumors have been measured (Figure 6B and 6C). Compared with the negative control group, the average tumor volume of tumors increased at slower rate in the sh-IGFBP2 group (Figure 6B). Furthermore, the average weight of tumors decreased in the sh-IGFBP2 group compared to the negative control group (Figure 6C). The immunohistochemistry staining results showed that a biomarker of proliferation (Ki-67) was suppressed in the sh-IGFBP2 group (Figure 6D).

Gemcitabine is still as a first-line chemotherapeutic agent of PDAC [27]. To determine if IGFBP-2 knockdown enhance sensitivity to chemotherapeutic drugs, the sh-IGFBP2 Bxpc-3 and CFPAC cells were exposed to increasing concentrations of gemcitabine (Bxpc-3 cells exposed to 0-10 μM, CFPAC cells exposed to 0-100 μM). The CCK-8 assay is a measure of the capability of reduction, which may reflect viability or proliferation. The cell viability was determined using a CCK-8 assay. As shown in Figure 4C, knockdown of IGFBP-2 significantly reduced cell viability in Bxpc-3 and CFPAC cell lines after 72 h treatment. Thus, our data showed that silencing IGFBP-2 inhibits proliferation and enhances sensitivity to gemcitabine. On the basis of these results, an orthotopic tumor model was developed to further assess the tumor-promoting effect of IGFBP-2 and its *in vivo* effect on the sensitivity to gemcitabine. The animals were evaluated once a week using the small animal imaging system and the volume of tumors was recorded at 35 days (Figure 6A). The bioluminescence imaging results demonstrated that the tumor volume of control groups gradually increased with the passage of time compared with the treatment groups. The immunohistochemistry staining results showed that Ki-67 was further reduced in the sh-IGFBP2 + GEM group (Figure 6D). These data demonstrated that knockdown of IGFBP-2 inhibits tumor proliferation and increases sensitivity to gemcitabine both *in vitro* and *in vivo*.

## DISCUSSION

IGFBP-2 is composed of 328 amino acid residues and is 36 kDa in size. IGFBP-2 contains RGD and HBD motifs which can directly bind to integrins and extracellular matrix and induce diverse biological actions independent of IGFs. The tumor suppressive functions of IGFBP-2 align with its ability to bind IGFs, while its oncogenic properties appear to be IGF-independent [28]. IGFBP-2 requires many different IGF/IGFR independent signaling pathways to perform its actions, including p53 [29], PTEN and PI3K/Akt [30] and MMPs [31]. IGFBP-2 also has effects on integrin and integrin-linked kinase (ILK) function [32] as well as promotion of angiogenesis through stimulating VEGF [33].

IGFBP-2 is being evaluated as a potential cancer biomarker. IGFBP-2 is highly expressed in tissues undergoing rapid cell division and motility. Several studies have reported that it is an important regulator of cell invasion and migration [33–36]. It has been established that enhanced expression of IGFBP-2 is associated with the progression of tumorigenesis in gliomas [33], prostate cancer [34] and breast cancer [35]. Overexpression of IGFBP2 enhances cell motility and induces liver metastasis in colorectal cancer through the L1 neuronal cell adhesion receptor [36]. Furthermore, a high level of IGFBP-2 is associated with higher risk of peripancreas lymph node metastasis and it has been identified as an independent prognostic factor in pancreatic cancer [37]. In addition, IGFBP-2 has received an increasing amount of attention for its function in the maintenance, growth, and migration of PDAC [38]. IGFBP-2 inhibited pancreatic cancer cell proliferation *in vitro*. In established orthotopic pancreatic tumors, we found that knockdown of IGFBP-2 led to smaller tumor size, lighter tumor weight, and decreased the expression of Ki-67 in immunohistochemical analysis. Our results from wound healing and Transwell invasion assays indicated that knockdown of IGFBP-2 markedly inhibites pancreatic cancer cells migration and invasion compared with the negative control group. In addition, IGFBP-2 promoted distant metastasis of pancreatic cancer cells in the orthotopic nude mouse model.

IGFBP-2 is a critical point in the crosstalk of several signaling pathways. IGFBP-2 has been linked to Hedgehog signal activation expression in glioblastoma, prostate, breast, and hematopoietic tumors [29–31]. EMT is a process defining the progression that cells lose their polarized epithelial character and acquire a migratory mesenchymal phenotype [39]. IGFBP-2 has a significant role in the migration and invasion of various malignancies [40]. Gao et al suggested that IGFBP-2 is a key EMT inducer for PDAC [13]. What’s more, the Hedgehog signaling pathway is associated with the EMT in pancreatic cancer [27]. PTCH1, the critical molecular of the Hedgehog pathway, is itself also the target gene of GLI1 [25]. The activated GLI1 transcription factors translocate to the nucleus and promote transcription of IGFBP-2 [26]. Therefore, we focused on the interaction between IGFBP-2 and PTCH1. Silencing of PTCH1 decreased mesenchymal markers (E-cadherin) and increased epithelial markers (vimentin and N-cadherin). Furthermore, silencing PTCH1 did not enhance the effect of IGFBP-2 in the EMT process. Interestingly, silencing IGFBP-2 decreased the expression of PTCH1, but had no effect on Gli1 expression. Overexpression of PTCH1 prevents the impact of IGFBP-2 on EMT. Our data indicated that IGFBP-2 exhibits oncogenic activity and induces EMT partially through Hedgehog pathway by inhibiting the expression of PTCH1.

IGFBP-2 possesses intrinsic activities occurring independently of IGFs, which is crucial for the activation of cellular mechanisms involved in regulation of resistance to chemotherapeutics. Several studies have reported the critical roles of IGFBP-2 in resistance to chemotherapy. IGFBP-2 is critical for chemoresistance in acute lymphoblastic leukemia cells [20]. Overexpression of IGFBP2 has been associated with resistance to paclitaxel [41] and antihormone therapy in breast cancer [42]. IGFBP2 is causally associated with dasatinib resistance and is used as a biomarker for the identification of dasatinib responders among patients with lung cancer [23]. Moreover, exogenous IGFBP-2 has been shown to exhibit chemoresistance to TMZ in glioma cells [19]. Silencing IGFBP-2 expression reduces the resistance to docetaxel in prostate cancer cells [18] and results in significant sensitization to cisplatin in esophageal adenocarcinoma cells [15]. Although gemcitabine has been used as a first-line therapy for PDAC, the management is extremely poor due to lack of an efficient therapy and development of chemoresistance. Effort has been made to develop new agents or strategies against therapy resistant pancreatic cancer. Due to increased IGFBP-2 expression levels reported in many malignancies and the link of IGFBP-2 to chemoresistance, targeting IGFBP-2 may increase the efficacy of gemcitabine. Our study showed that the sensitivity to gemcitabine was significantly enhanced after IGFBP-2 knockdown in pancreatic cancer cells. Moreover, knockdown of IGFBP-2 increased the anti-tumor properties of gemcitabine in the orthotopic tumor model. Given these results, we conclude that inhibition of IGFBP-2 would enhance the sensitivity of pancreatic cancer cells to gemcitabine. This study provides hope for the development of new therapies to block tumor growth by enhancing the efficacy of gemcitabine.

Knockdown of IGFBP-2 suppressed the proliferation, invasion and metastatic properties of pancreatic cancer cells, and it also increased the sensitivity to gemcitabine. Our data suggests that IGFBP-2 might be a potential therapeutic and offers a promising therapeutic approach for the development of more effective targeted therapies in the treatment of PDAC patients.

## MATERIALS AND METHODS

### Materials

Gemcitabine was purchased from Eli Lily (France). The following antibodies were used in this study: IGFBP-2, PTCH-1and Gli1 (Santa Cruz Biotechnology, CA, USA); E-cadherin, N-cadherin and vimentin (Cell Signaling Technology, Inc., MA, USA); and Ki-67 (Abcam Inc., MA, USA).

### Cell culture

The human pancreatic cancer cell lines Bxpc-3 and Panc-1, as well as human pancreatic duct epithelial (HPDE) cells were purchased from the American Type Culture Collection (Manassas, USA). Aspc-1, CFPAC and SW1990 cells were purchased from the Type Culture Collection of the Chinese Academy of Sciences (Shanghai, China). Aspc-1 and Bxpc-3 cells were cultured in RPMI 1640 medium (HyClone, USA), and CFPAC, Panc-1, SW1990 and HPDE cells were cultured in Dulbecco's Modified Eagle's Medium (Gibco, USA). All media were supplemented with 10% fetal bovine serum (Gibco, USA), 1% penicillin and streptomycin, and all cells were cultured at 37°C in humidified air with 5% CO_2_.

### Transient transfection

siRNA kncockdown of IGFBP-2 and PTCH1 was performed as previously described [43]. siIGFBP2-1, siIGFBP2-2, siIGFBP2-3, siPTCH1-1, siPTCH1-2, siPTCH1-3 and negative control siRNA (NC) were obtained from RiboBio (Guangzhou, China). Briefly, Bxpc-3 and CFPAC cells were plated onto a six-well plate and allowed to adhere overnight. Then, the pancreatic cancer cells were transfected with each siRNA using the riboFectTM CP transfection kit (RiboBio) according to manufacturer’s instructions for 48 hour used for subsequent experiment. There sequences were listed as follows:

si-NC CGUACGCGGAAUACUUCGAdTdT

si-IGFBP2-1 CGATGACCACTCAGAAGGA

si-IGFBP2-2 GTGTCATCTCTTCTACAAT

si-IGFBP2-3 CCTGTACAACCTCAAACAG

si-PTCH1-1 GCGCTGTCTTCCTTCTGAA

si-PTCH1-2 GCCAAACAATTACAAGAAT

si-PTCH1-3 CCAGCCGTGTCCATGTATA

### Plasmid construction and transfection

The IGFBP-2 plasmid and mock vector were purchased from Youbio Biological Technology (Hunan, China). For plasmid transfection, the cells were seeded in 6-well plates and 3μg of plasmids were transfected with Lipofectamine 2000 (Invitrogen). Mock vector was used as a negative control. After transfection for 48h, the cells were collected for Western blots assays. The efficiency of IGFBP-2 overexpression was evaluated by qRT-PCR assays and Western blot assays.

### Lentiviral infection

Human Lenti-shIGFBP-2-GFP and Lenti-shcon were designed and purchased from GeneChem Technologies (Shanghai, China). The transfection was performed according to standard procedures. Following lentiviral infection, single-cell clonal isolates were selected in the presence of presence of puromycin for 2 to 4 weeks, qRT-PCR assays and Western blot assays were used to validate the stably transfected cells.

### Cell proliferation assay

Cell proliferation was monitored by a CCK-8 kit according to the manufacturer’s instructions. Negative control and sh-IGFBP2 cells (3 ×10^3^) were seeded into 96-well plate and the proliferation was evaluated at 0, 24, 48 and 72 h. The absorbance was measured at 450 nm using a microplate reader (Bio Tek, USA).

### Cell viability assay

Cell viability was performed as described previously [43]. Cells were plated at a density of 3–5×10^3^ cells/well with 200 μl of medium in 96-well microplates with increasing doses of gemcitabine (Bxpc-3 cells exposed to 0-10 μM, CFPAC cells exposed to 0-100 μM). After treatment, CCK-8 solution (10 μl) was added to each well and the plates were incubated at 37°C for 90 min. The absorbance of the cell suspension was measured with a microplate reader at a wave length of 450 nm. 200μl medium containing 20 μl CCK-8 solution was served as control.

### Colony formation assay

The colony formation assay was performed as described previously [44]. Transfected sh-NC and sh-IGFBP2 cells were seeded at a density of 0.5 ×10^3^ per well and the medium was changed every three days. After 2 weeks, the colonies were counted after fixation in 4% paraformaldehyde for 30 min with 1% crystal violet staining. The colonies were counted manually in five fields (10×, Olympus, Japan).

### Immunofluorescence (IF)

For immunofluorescence of cultured cells, Bxpc-3 and CFPAC cells were fixed with 4% paraformaldehyde, permeabilized with 0.5% Triton X-100 and blocked in 1% BSA in PBS 48 h after transfection. After incubation with the primary antibody as indicated overnight at 4°C, the cells were washed three times with PBS, and cells were incubated with secondary antibodies (Beyotime, China) for 1 h at room temperature. Finally, 4', 6-diamidino-2-phenylindole (DAPI, Beyotime, China) was added to stain the cell nuclei and images were captured by a Leica DMRA fluorescence microscope (Rueil-Malmaison).

### Wound healing assay, migration and invasion assays

Cells were cultured in 6-well plates and allowed to form a confluent monolayer for 24 h. The monolayer was scratched with a sterile pipette tip (200 μl), and then washed twice with PBS and incubated in serum-free medium and mitomycin. Images were taken at 0h, 24h and 48 h. The wound areas were photographed with a microscope (10×, Olympus, Japan). The percentage of wound closure was estimated by Image J software.

Migration and invasion assays were performed as described previously [45]. The cells were plated in 24-well BioCoat Matrigel Invasion Chambers (Corning, Manassas, VA, USA). The cells on the upper surface of the filter were carefully removed with a cotton swab after 48 h. The cell numbers on each membrane were counted in five high-power fields using a microscope (20×, Olympus, Japan).

### Western blot assay

Western blot analysis was performed as described previously [44–46]. In brief, cells were sonicated in RIPA buffer and homogenized after being washed twice in PBS. Debris was removed by centrifugation at 12000×g for 10 min at 4°C, and protein concentration was determined using the BCA protein assay. Whole-cell lysates with approximately 40 μg of proteins were resolved on 10% SDS-PAGE and subjected to Western blot assays. After incubation with appropriate secondary antibody and washing, the bands were visualized using an enhanced chemiluminescence (ECL) kit followed by exposure of the membrane to X-ray film. β-actin was used as a loading control.

### Quantitative real-time polymerase chain reaction (qRT-PCR)

qRT-PCR was performed as previously [47]. The cDNA was synthesized from total RNA using the TOYOBO Kit (Osaka, Japan). qRT-PCR was performed on the Applied Biosystem 7500 with SYBR Green (Roche, USA). The relative expression levels of mRNA were calculated and quantified using the 2 ^–ΔΔCT^ method after normalization for the expression of the control, and the expression of GAPDH served as the endogenous control. The IGFBP-2, PTCH1 and GAPDH primer sequences were as follows: IGFBP-2 forward (5’ to 3’): AGGAGACTTAATGGACGCTTGT, reverse (5′ to 3′): GCTCCTTCATACCCGACTTGAG; PTCH1 forward (5’ to 3’): TCCAGGCAGCGGTAGTAGTAGTGGT, reverse (5′ to 3′): GCTGTAGCGGGTA TTGTCGTGT; GAPDH forward (5′ to 3′): CTCTGCTCCTCCTGTTCGAC, reverse (5′ to 3′): GCGCCCAATACGACCAAATC.

### Orthotopic tumor model and treatments

The orthotopic tumor models were established according to the methods which we have described previously [48]. Animal care was performed in accordance with institutional guidelines and all animal experiments were done using protocols approved by the Institutional Review Board of the First Affiliated Hospital of Harbin Medical University. The female nude BALB/c mice (4-6 weeks old), were purchased from the Shanghai Experimental Animal Center of the Chinese Academy of Sciences (Shanghai, China). The mice were injected with Bxpc-3-Luc cells which were transfected with sh-IGFBP2 and the negative control vector. A total of 5×106 cells in 200 μl PBS were injected into the left flank of nude mice. Two weeks after injection, the primary sh-IGFBP2 and negative control group tumors were harvested and cut into 1 mm^3^ pieces. After two groups of mice were anesthetized with intraperitoneal injections of 0.5% pentobarbital (100 g/ml), the pieces of tumors from different groups were translocated into the pancreatic tail and fixed by 5-0 Prolene sutures.

The 28 mice were randomly assigned to four groups as follows: (a) negative control; (b) sh-IGFBP2; (c) negative control and gemcitabine and (d) sh-IGFBP2 and gemcitabine. The mice were injected intraperitoneally with gemcitabine (100mg/kg) twice weekly for 4 subsequent weeks. The mice were injected intraperitoneally 100mg/kg the D-luciferin (Xenogen, Hopkinton, MA), and the bioluminescence IVIS Imaging System (Berthold Technologies, Germany) was used to capture images of pancreatic tumors weekly. Body weights of mice were measured before the treatment every week. Changes in tumor growth and sites of metastasis were also evaluated. Mice were euthanized at day 35, the pancreas was excised, tumors were weighed, and volume was measured for between group comparisons. The numbers of visible metastatic nodules more than 1mm3 in the gut, mesentery and spleen were counted.

### Immunohistochemical staining

The paraffin-embedded tissue sections (5 μm) were immunostained with E-cadherin, N-cadherin, vimentin, IGFBP2 and Ki-67 antibodies. Tissue sections were deparaffinized in xylene and rehydrated with ethanol. Tissue sections were then preincubated with 10% normal goat serum in PBS followed with incubation with primary antibody overnight at 4°C. Tissue sections were then stained with biotinylated secondary antibody (Vector lab, USA) for 1 hour at room temperature, followed by incubation with the Vectastain Elite ABC reagent (Vector lab, USA) for 30 min. The peroxidase reaction was developed with diaminobenzidine (DAB kit; Vector lab) and the slides were counterstained with hematoxylin (Sigma). The number of positive cells was counted in five high-power fields using a microscope (Olympus, Japan).

### Statistical analysis

The growth patterns of tumors were compared using the analysis of variance (ANOVA) test. Other results were expressed as mean values ± standard deviation, and a Student’s *t*-test was used to evaluate statistical significance. The data are shown as the mean ± SD, and differences are considered significant when* *P*<0.05, **P< 0.01, ***P<0.001 and ns *P*>0.05.
